# A BiO‐Optimizing Site Targeted (BOOST) Approach to Periodontal Regeneration Through Local Doxycycline Prior to Surgery: A Randomized Clinical Trial

**DOI:** 10.1111/jre.13389

**Published:** 2025-01-27

**Authors:** Mario Aimetti, Giacomo Baima, Virginia Lorenzetti, Nargiz Aliyeva, Mario Bottone, Giulia Maria Mariani, Federica Romano

**Affiliations:** ^1^ Department of Surgical Sciences, C.I.R. Dental School University of Turin Turin Italy

**Keywords:** antimicrobials (local), inflammation, intrabony defects, periodontal diseases, periodontal regeneration

## Abstract

**Aim:**

To test a BiO‐Optimizing Site Targeted (BOOST) approach to periodontal regeneration by the adjunctive use of locally delivered doxycycline (DOX) 2 weeks prior to minimally invasive surgery in terms of clinical and radiographic outcomes at 1 year.

**Methods:**

For this randomized clinical trial, stage III/IV periodontitis patients presenting sites with intrabony defects and bleeding on probing (BoP+) after steps 1–2 of periodontal treatment were included. Sites were treated via subgingival instrumentation with or without a BOOST approach by local DOX. After 2 weeks, defects were accessed by minimally invasive surgical technique with xenograft and amelogenins. Primary (clinical attachment level [CAL] gain) and secondary (probing pocket depth [PPD] reduction, composite outcomes, radiographic bone defect fill) outcomes were assessed at 12 months.

**Results:**

Sixty patients completed the study (30 on each group). BOOST led to lower preoperative BoP (*p* < 0.001) and better wound healing after surgery (*p* = 0.027). Both groups showed clinical and radiographic improvements at 1 year, with significant differences in mean CAL gain (4.1 ± 1.9 vs. 3.2 ± 2.0 mm; *p* = 0.019) and PPD reduction (4.4 ± 1.8 vs. 3.6 ± 1.9 mm; *p* = 0.040) favoring the test group. BOOST group also achieved higher composite outcome measure (PPD ≤ 4 mm and CAL gain ≥ 3; 83.3% vs. 46.7%; *p* = 0.006), pocket closure (PPD ≤ 3 mm or 4 mm BoP−; 83.3% vs. 60.0%; *p* = 0.045), and defect fill (3.5 ± 1.2 vs. 2.7 ± 1.3 mm; *p* < 0.001) compared to the control group.

**Conclusion:**

A BOOST approach by local doxycycline 2 weeks before surgery enhanced the local inflammatory control prior to periodontal regeneration and post‐operative early wound healing, yielding to improved clinical and radiographic outcomes at 1 year.

**Trial Registration:**

ClinicalTrials.gov identifier: NCT05878353

## Graphical abstract

Localized pre‐surgical modulation of inflammation using doxycycline (BOOST) improved early wound healing and enhanced clinical and radiographic outcomes of periodontal regeneration at 1 year, demonstrating significant benefits in terms of CAL gain, defect fill, and pocket closure compared to standard care.

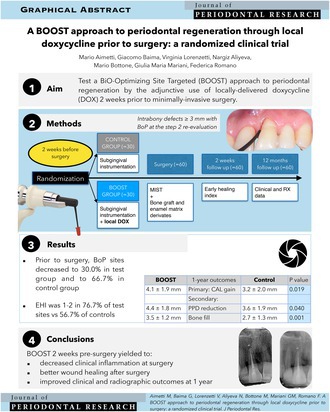

**Summary**

- Background
  ○ Persistent inflammation at the interproximal site level prior to periodontal regenerative surgery is regarded as factors negatively affecting the treatment success, although its impact has not been assessed in a randomized clinical trial to date.
- Added value of this study
  ○ A BiO‐Optimizing Site Targeted (BOOST) approach by local doxycycline with the scope of controlling the infective‐inflammatory status prior to periodontal regenerative surgery was tested, achieving significant results both in terms of soft tissue early wound healing and 1‐year clinical and radiographic performance.
- Clinical implications
  ○ Pre‐surgical modulation of the inflammatory status through a BOOST approach by local doxycycline may be considered in sites presenting deep bleeding on probing in order to improve the outcomes of periodontal regeneration.

## 1 Introduction

Clinical practice guideline from the European Federation of Periodontology (EFP) recommends periodontal regeneration surgery to treat teeth with residual pockets with probing pocket depth (PPD) ≥ 6 mm associated with intrabony defects of 3 mm or deeper [1, 2]. Site‐related factors, including defect morphology and soft tissue quality, as well as surgical technique and surgeon's skills, can influence the success of periodontal regeneration surgery [3, 4, 5, 6]. Indeed, the presence of sites with persistent bleeding on probing (BoP+) after step 2 of therapy has been linked to higher complication rate and suboptimal clinical outcomes following the regenerative treatment [4, 7, 8].

Efforts to enhance site‐specific infection and inflammation control during periodontal therapy may be supported by the subgingival application of local antimicrobials [2, 9]. Utilizing a local delivery system offers advantages over systemic prescription, allowing for a low‐dose delivery to the periodontal pocket with targeted action, minimized side effects, and reduced risk of bacterial tolerance [10]. While a recent systematic review demonstrated the short‐term benefits of local antimicrobials in conjunction with subgingival instrumentation (step 2 of therapy), their long‐term effect has been questioned leading to an open recommendation for clinical practice [9].

Doxycycline hyclate carried with a biodegradable polylactic‐polyglycolic acid gel (DOX) has been proposed as adjuvant therapy in treating residual pockets due to its antimicrobial properties and impact on matrix metalloproteinases (MMPs) [9, 11, 12]. Indeed, its ability to bind zinc and calcium ions within the catalytic domain results in the inhibition of collagenase enzymes and a reduction in inflammatory cytokines in the gingival crevicular fluid [13, 14, 15]. This may contribute to decreased connective tissue and bone resorption, ultimately improving healing outcomes in periodontal therapy.

The underlying rationale of this study is that a BiO‐Optimizing On‐Site Targeted (BOOST) approach involving the subgingival application of DOX to selectively modulate the immune‐inflammatory conditions in the microenvironment prior to periodontal regeneration would lead to better interproximal soft tissue wound healing and final results. Therefore, the primary aim of this trial was to test the BOOST approach by local DOX administered 2 weeks prior to surgery on the clinical and radiographic outcomes of periodontal regeneration. The secondary aim was to assess the Early Wound Healing Index (EHI) at 2 weeks after surgery and relate it to the final study outcomes.

## 2 Methods

The Institutional Ethical Committee of the A.O.U. Città della Salute e della Scienza of Turin (Italy) granted approval for the protocol under the reference number 00309/2021. Adherence to the principles outlined in the Declaration of Helsinki and reporting in accordance with the CONSORT statement underscore the ethical and transparent conduct of this study [16]. Informed consent was obtained from all patients.

### 2.1 Study Design and Population

This investigation employed a triple‐blind, randomized clinical trial design with two parallel groups and an allocation ratio of 1:1. Consecutive patients who underwent steps I–II of periodontal treatment at the Section of Periodontology, C.I.R. Dental School, University of Turin, were meticulously screened for eligibility between September 2022 and June 2023. Prior to screening for potential enrolment, all patients underwent steps I–II of periodontal treatment according to EFP guideline [1], with subgingival instrumentation performed with a quadrant‐wise approach without the use of local or systemic adjunctives. After a period of 8–12 weeks, patients were re‐evaluated for inclusion into the study. Inclusion criteria comprised the following: (1) a diagnosis of stage III or IV periodontitis [17]; (2) full‐mouth plaque score (FMPS) and full‐mouth bleeding score (FMBS) < 15% at the time of enrollment; (3) presence of one tooth with residual PPD ≥ 6 mm, BoP+, and a radiographic intrabony component ≥ 3 mm, suitable for a minimally invasive surgical technique (MIST) procedure [18]. This latter criterion was evaluated by a bone sounding procedure under anesthesia at the time of the presurgical procedure suggesting the presence of an intrabony defect extending to both the buccal and lingual/palatal surface of the affected tooth.

Exclusion criteria were (1) age < 18 years; (2) smoking subjects; (3) systemic or local contraindications for periodontal surgery; (4) systemic diseases affecting periodontal healing (i.e., diabetes mellitus, immune deficiencies); (5) pregnancy and lactation; (6) presence of furcation involvement at the affected tooth; (7) history of periodontal surgery at the experimental teeth; (8) allergies to DOX and tetracyclines; and (9) assumption of local and systemic antimicrobials in the last 3 months.

### 2.2 Sample Size and Randomization

Difference in CAL gain between test and control groups was set as primary outcome of the study. No previous study employed the same design; therefore, sample size was inferred indirectly. Based on the expected mean CAL gain and standard deviation of periodontal regeneration with a combined approach (4.1 ± 0.8 mm) [1, 19] and the adjunctive effect of DOX reported in a previous trial (0.5 mm) [20], a sample size of 51 patients was needed at 0.05 two‐sided alpha error and 80% power. Considering a drop‐out rate of 20%, 60 patients (30 in the test and 30 in the control group) were considered.

A random permuted blocks randomization list with a 1:1 allocation ratio was generated using a statistical software (SPSS, IBM version 28) by an independent researcher, not involved in the selection, treatment, outcomes assessment, and statistical analysis. To conceal assignment, forms with the treatment modality were put into opaque and sealed envelopes with the patient number on the outside. The envelopes were placed into the custody of the study coordinator, who opened them prior to the eventual DOX application (2 weeks before surgery) and informed the clinician. The patient, the surgeon, and the examiner who performed the measurements were blinded to treatment assignment.

### 2.3 Intervention

#### 2.3.1 Pre‐Surgical Procedures

Experimental treatments were performed between January and July 2023. Two weeks before surgery, each experimental site received subgingival debridement alone (control) or in combination with a BOOST approach by a single local administration of DOX (test; Ligosan, Kulzer, Germany). Debridement was performed under local anesthesia (mepivacain 2% 1:1000) by mean of ultrasonic devices with ultrathin tips by the same experienced clinician. Great attention was made to avoid marginal and interproximal soft tissue damage. In the test group, local DOX was administered according to the manufacturer's instruction. After rinsing and drying the root surface, the plastic needle of the syringe was inserted into the sulcus and the product was progressively released in the periodontal pocket up to the gingival margin. The syringe was then removed and a cotton pellet was used to compact the gel into the sulcus. In the group receiving gentle debridement alone, the whole procedure related to the administration of local DOX was simulated without injecting the antibiotic. The net operative time for the procedure was approximately 5 min. Patients of both arms were then instructed to make a gentle supragingival use of interdental brushes in the study area for the following 7 days.

#### 2.3.2 Surgical Procedure

The experimental sites were later approached by means of an MIST [18] under magnification loops by III‐year postgraduate residents at the Section of Periodontology. Two senior clinicians (MA and GMM) with long‐term experience with this technique directly supervised the clinical interventions. Briefly, vertical‐releasing incisions were avoided, and the full‐thickness flap was minimally raised both on the buccal and lingual/palatal surfaces [21]. Granulation tissue was removed from the defects [22], and the root was thoroughly scaled using minicurettes and magnetostrictive ultrasonic device with extended thin shank narrowing to a probe‐sized tip. After chemical treatment with EDTA (PrefGel, Institut Straumann AG), all regenerative procedures were carried out using a combination of enamel matrix derivatives (Emdogain, Institut Straumann AG) and bone xenograft (Bio‐Oss, Geistlich Pharma AG) [1, 2, 3, 23]. The flaps were passively repositioned and sutured (Gore‐tex, 6/0 WL Gore & Associated, Flagstaff, AZ, USA).

#### 2.3.3 Postoperative and Maintenance Care

All patients received analgesic medication (ibuprofen 600 mg, every 12 h for 2 days) and 0.12% chlorhexidine digluconate mouthrinse for 1 min (2 times/day for 2 weeks) [24]. Clinical check and wound cleaning with chlorhexidine were performed 1 week later. During this postoperative period, patients were prescribed to avoid toothbrushing and flossing in the treated area. Sutures were removed 14 days after surgery. After suture removal, the patient was instructed to use a soft toothbrush for an additional 2 weeks. Following this period, the patient resumed their normal oral hygiene routine, which also included the use of interproximal cleaning aids. Patients were then recalled at 1, 3, 6, and 9 months after surgery, when oral hygiene instructions were reinforced and supragingival instrumentation provided.

### 2.4 Clinical and Radiographic Measurements

Clinical measurements were taken at the deepest point of the selected defects by using a manual 1‐mm graduated periodontal probe (PCP‐UNC 15, Hu‐Friedy, Chicago, IL) at baseline (14 days before surgical treatment, T0), at the day of surgery (T1), and at the completion of the 1‐year follow‐up by the same blinded examiner (GB). The following clinical parameters were assessed at T0 and T1: presence/absence of dental plaque (PI), presence/absence of BoP, PPD, gingival recession (REC), CAL, width of keratinized tissue (KT), and gingival phenotype (by assessing probe transparency). To perform the intra‐examiner calibration, 10 non‐study patients presenting with intrabony defects were evaluated by the examiner on two separate occasions within 48 h and the intra‐class correlation coefficient (ICC) was calculated. The intra‐rater agreement was 0.92 (95% CI: 0.80, 0.97) for CAL and 0.95 (95% CI: 0.87, 0.98) for PPD, showing an almost perfect agreement for both the variables. The number of bony walls was assessed and registered intra surgically. Two weeks after surgery, the EHI [25] was assessed by a second blinded assessor (MA). Intra‐examiner calibration was performed after training with another clinician (GMM) on 10 calibration rounds, until a Cohen kappa score > 0.8 was reached.

Periapical radiographs were taken at baseline and 12 months postoperatively using the long‐cone paralleling technique and with a customized support made of impression material to standardize the approach. The radiographs were analyzed using a software program (ImageJ, National Institutes of Health) by a third masked investigator (VL). The intra‐examiner reproducibility was assessed by measuring 15 radiographs from teeth not involved in the study and the *k*‐score was assessed. The measurements were carried out twice within a 14‐day interval, revealing an agreement of 92% within ± 1 mm for the linear measurements and 94% for an angle range of ± 1°. ICC was 0.86 (95% CI: 0.67, 0.94). The intrabony defect depth (IBD) was recorded as the distance between the alveolar bone crest (BC) and the bottom of the defect (BD). Intra‐osseous defect angle was determined by the line defined by the root surface of the involved tooth and the line defined by the bone wall of the defect. The radiographic bone fill and the bone defect angle change were calculated by subtracting the measurement recorded at the 12‐month postoperative examination from the baseline [26].

### 2.5 Outcome Measures

The primary outcome measurement of the study was CAL gain at 1‐year recall visit. Secondary outcome measurements included: (1) PI; (2) BoP; (3) PPD reduction; (4), REC changes; (5) proportion of cases with pocket closure (PPD ≤ 3 mm or 4 mm with no BoP) [27]; (6) proportion of successfully treated cases (final PPD ≤ 4 mm and CAL gain ≥ 3 mm) as evaluated according to the composite outcome measure (COM) for regenerative treatment of intrabony defects [28]; (7) radiographic bone change; (8) radiographic defect angle change.

### 2.6 Statistical Analysis

Statistical analyses were conducted using commercially available software (SPSS, IBM version 28). The statistical unit was the patient. To test whether the data were normally distributed the Shapiro–Wilk test was done. Differences in the frequency of the categorical variables were verified using the chi‐squared test or the Fisher exact test. Unpaired *t*‐test and Mann–Whitney *U*‐test were used to assess the significance of difference between the two groups with Bonferroni correction for multiple comparisons. Friedman's test was used to detect intragroup differences in clinical parameters over time, followed by pairwise tests (Dunn test). Paired *t*‐test was applied to radiographic outcomes. For the comparison of pocket closure and COM between the two approaches, the chi‐squared test was employed. An explorative multivariate linear regression model was constructed testing the effect of independent variables (age, treatment allocation, defect configuration, EHI, baseline PPD, biotype, baseline radiographic angle) on CAL gain at 1 year.

## 3 Results

### 3.1 Patient Characteristics

**FIGURE 1:**
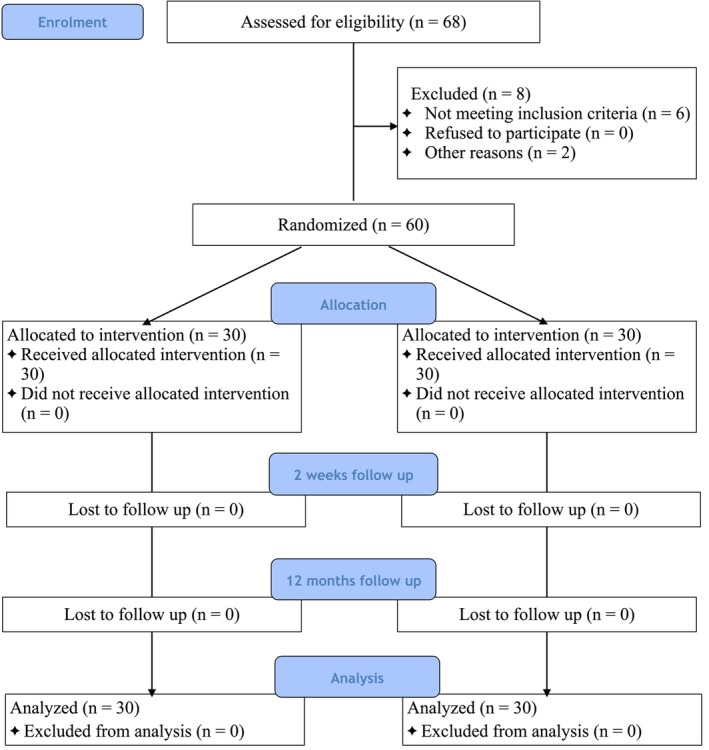
CONSORT flowchart of the study design.

Figure 1 summarizes the flow chart of the study. Sixty patients were enrolled in the study. All participants, 30 in the test group (14 females; mean age: 58.3 ± 10.6 years) and 30 in the control group (17 females, mean age: 58.0 ± 11.1 years) received the allocated procedure, completed the 1‐year examination, and were included in the statistical analyses. Baseline (T0) characteristics for test and control groups at patient‐ and site‐level are reported in Table 1. In the test group, 17 patients had PPD 6–7 mm, and the remaining 13 had PPD ≥ 8 mm. The corresponding figure for the control group was 14 and 16 patients, respectively (*p* = 0.790).

**TABLE 1:** Characteristics of patients and treated sites.

	BOOST (*n* = 30)	Controls (*n* = 30)
Age (years; mean ± SD)	58.3 ± 10.6	58.0 ± 11.1
Females/males (*n*)	14/16	17/13
FMPS (%; mean ± SD)	11.3 ± 2.3	10.6 ± 1.7
FMBS (%; mean ± SD)	9.9 ± 1.9	9.5 ± 1.9
Tooth type (anterior/premolar/molar) (*n*)	11/13/6	11/9/10
Gingival phenotype (thin/thick) (*n*)	8/22	7/23
Buccal keratinized tissue width (mm; mean ± SD)	4.5 ± 2.1	4.6 ± 1.4
Main defect configuration (1‐/2‐/3‐wall)	3/15/12	3/9/18

Abbreviations: BOOST, BiO‐Optimizing Site Targeted approach by local doxycycline (test group); FMBS, full‐mouth bleeding score; FMPS, full‐mouth plaque score; SD, standard deviation.

### 3.2 Clinical Outcomes

#### 3.2.1 BoP Reduction Before Surgery and EHI

No adverse outcomes related to the study procedures were observed in any of the patients. At T1, there were no intragroup changes in any of the clinical periodontal parameters compared to T0, except for BoP (Table 2). This latter variable decreased to 30.0% in the test group, while in the control group, it decreased to 66.7% (*p* < 0.001).

**TABLE 2:** Site‐level clinical measurements at baseline, surgery, and 12 months.

Clinical measurements	Baseline	Surgery	*p* (baseline to surgery) intra‐group	12 months	Δ baseline to 12 months	*p* (baseline to 12 months) intra‐group	*p* (surgery to 12 months) intra‐group
PI (%)							
BOOST	30.0	10.0	> 0.05^a^	20.0	10.0	> 0.05^a^	> 0.05^a^
Control	36.7	13.3	> 0.05^a^	23.3	13.4	> 0.05^a^	> 0.05^a^
*p* inter‐group	> 0.05^b^	> 0.05^b^		> 0.05^b^	> 0.05^b^		
BoP (%)							
BOOST	100.0	30.0	**< 0.001** ^**a**^	26.7	73.3	**< 0.001** ^**a**^	> 0.05^a^
Control	100.0	66.7	**< 0.001** ^**a**^	33.3	66.7	**< 0.001** ^**a**^	**0.021** ^**a**^
*p* inter‐group	> 0.05^b^	0.004^b^		> 0.05^b^	> 0.05^b^		
CAL (mm; mean ± SD)							
BOOST	9.5 ± 2.6	9.4 ± 2.5	> 0.05^c^	5.4 ± 1.7	4.1 ± 1.9	**< 0.001** ^**c**^	**< 0.001** ^**c**^
Control	9.7 ± 2.2	9.5 ± 2.4	> 0.05^c^	6.5 ± 1.8	3.2 ± 2.0	**< 0.001** ^**c**^	**< 0.001** ^**c**^
*p* inter‐group	> 0.05^d^	> 0.05^d^		**< 0.001** ^**d**^	**0.019** ^**d**^		
PPD (mm; mean ± SD)							
BOOST	7.7 ± 1.7	7.4 ± 1.6	> 0.05^c^	3.3 ± 0.7	4.4 ± 1.8	**< 0.001** ^**c**^	**< 0.001** ^**c**^
Control	7.9 ± 1.9	7.5 ± 2.1	> 0.05^c^	4.3 ± 0.6	3.6 ± 1.9	**< 0.001** ^**c**^	**< 0.001** ^**c**^
*p* inter‐group	> 0.05^d^	> 0.05^d^		**0.025** ^**d**^	**0.040** ^**d**^		
Recession (mm; mean ± SD)							
BOOST	1.9 ± 1.7	2.0 ± 1.6	> 0.05^c^	2.1 ± 1.7	0.2 ± 0.9	> 0.05^c^	> 0.05^c^
Control	1.8 ± 1.2	1.9 ± 1.3	> 0.05^c^	2.2 ± 1.4	0.4 ± 0.7	**0.039** ^**c**^	> 0.05^c^
*p* inter‐group	> 0.05^d^	> 0.05^d^		> 0.05^d^	0.497^d^		

*Note:* Statistically significant difference are highlighted in bold at 0.05 level.

Abbreviations: BOOST, BiO‐Optimizing Site Targeted approach by local doxycycline (test group); CAL, clinical attachment level; PPD, probing pocket depth; SD, standard deviation.

^a^ McNemar test.

^b^ Chi‐squared test.

^c^ Dunn test (when Friedman test statistically significant).

^d^ Mann–Whiney *U*‐test with Bonferroni correction.

At T2, the EHI had an average value of 1.5 ± 0.9 in the test group, while in the control group, the average value was 2.2 ± 1.4 (*p* = 0.041). Among patients receiving BOOST, the EHI was 1–2 in 23 subjects (76.7%) and 3–5 in 7 (23.3%). Among controls, the EHI was 1–2 in 17 patients (56.7%) and 3–5 in 13 (43.3%).

#### 3.2.2 Clinical Parameters at 1 Year

The clinical measurements of the sites included in the two treatment groups at baseline and 12 months are presented in Table 2. Site‐level PI slightly reduced in both groups without statistical significance, whereas BoP significantly decreased from baseline to 12 months (both *p* < 0.001). Both groups demonstrated statistically significant improvements in CAL and PPD compared to their baseline measurements at 12 months (*p* < 0.001). A CAL gain of 4.1 ± 1.9 mm, a mean PPD reduction of 4.4 ± 1.8 mm, and an REC increase of 0.2 ± 0.9 mm were observed at 12 months in the BOOST group. The corresponding clinical measurements in the control group were 3.2 ± 2.0 mm of CAL gain (*p* = 0.019), 3.6 ± 1.9 mm of mean PPD reduction (*p* = 0.040), and 0.4 ± 0.7 mm of REC increase (*p* = 0.497).

In the multiple linear regression model (Table 3), CAL gain was significantly associated with treatment (*p* < 0.001), defect morphology (*p* = 0.003), EHI (0.008), and PPD at baseline (p < 0.001).

**TABLE 3:** Multiple linear regression models showing the relationships between clinical predictors and CAL gain at 12 months.

Variable	*β*	95% CI	*p*
Treatment (BOOST vs. control)	1.12	0.56 to 1.68	< 0.001
Bony walls (contenitive vs. non contenitive)	0.98	0.35 to 1.60	0.003
EHI (1–2 vs. 3–4–5)	0.93	0.26 to 1.61	0.008
PPD baseline (mm)	0.71	0.53 to 0.90	< 0.001
Intercept	−3.56	−4.86 to −2.27	< 0.001

Abbreviations: BOOST, BiO‐Optimizing Site Targeted approach by local doxycycline (test group); CI, confidence interval; EHI, early healing index; PPD, probing pocket depth.

Frequency distribution of pocket closure and COM in both groups are displayed in Table 4. Pocket closure (PPD ≤ 3 mm or 4 mm with no BoP) in the BOOST group was observed in 25 of 30 sites (83.3%). Conversely, in the control group, pocket closure was observed in 18 of 30 sites (60.0%) (*p* = 0.045). Number needed to treat (NNT) for achieving one additional study endpoint was 4.29. Concerning COM results (PPD ≤ 4 mm and CAL gain ≥ 3 mm), 25 of 30 sites (83.3%) in the BOOST group and 14 of the 30 sites (46.7%) in the control group fulfilled the criteria of successful outcome (*p* = 0.006). NNT for obtaining one unit of advantage by using COM outcome was 2.73.

**TABLE 4:** Pocket closure and composite outcome measure (COM) at 12 months.

	*N* (%)
Pocket closure (PPD ≤ 3 or 4 mm with no BoP)	
BOOST	25 (83.3)
Control	18 (60.0)
*p* inter‐group	**0.045** ^**a**^
COM (PPD ≤ 4 mm and CAL gain ≥ 3 mm)	
BOOST	25 (83.3)
Control	14 (46.7)
*p* inter‐group	**0.006** ^**b**^

Abbreviations: BOOST, BiO‐Optimizing Site Targeted approach by local doxycycline (test group); CAL, clinical attachment level; PPD, probing pocket depth; SD, standard deviation. Statistical significant differences are highlighted in bold.

^a^ Chi‐squared test.

^b^ Fisher exact test.

### 3.3 Radiographic Outcomes

Radiographic measurements at baseline and 12 months are summarized in Table 5. Statistically significant improvements in terms of IBD and defect angle in both groups were observed (*p* < 0.001). Radiographic IBD demonstrated a reduction of 3.5 ± 1.2 mm in the BOOST group and 2.7 ± 1.3 mm in the control group (*p* < 0.001). Defect angle change amounted to 9.7° ± 5.1° in the BOOST group and 3.6° ± 4.5° in the control group (*p* < 0.001).

**TABLE 5:** Radiographic measurements at baseline and 12 months.

Radiographic measurements	Baseline	12 months	^Δ^0–12 months	*p* intra‐group
BC–BD (mm; mean ± SD)				
BOOST	5.1 ± 1.3	1.7 ± 0.8	3.5 ± 1.2	**< 0.001** ^**a**^
Control	5.3 ± 2.0	2.6 ± 1.3	2.7 ± 1.3	**< 0.001** ^**a**^
*p* inter‐group	> 0.05^b^	**0.002** ^**b**^	**0.027** ^**b**^	
Defect angle (°)				
BOOST	30.7 ± 8.3	40.5 ± 8.9	9.7 ± 5.1	**< 0.001** ^**a**^
Control	31.8 ± 8.9	35.4 ± 8.7	3.6 ± 4.5	**< 0.001** ^**a**^
*p* inter‐group	> 0.05^b^	**0.032** ^**b**^	**< 0.001** ^**b**^	

Abbreviations: BC–BD, depth of the intra‐osseous defect; BOOST, BiO‐Optimizing Site Targeted approach by local doxycycline (test group); SD, Standard Deviation. Statistical significant differences are highlighted in bold.

^a^ Paired *t*‐test.

^b^ Independent samples *t*‐test.

## 4 Discussion

The present RCT aimed at evaluating the adjunctive effect of a BOOST approach by local DOX administered 2 weeks prior to surgery with the scope of reducing the local inflammatory burden on the outcomes of periodontal regeneration. Short‐term results showed a significant decrease of BoP in the experimental sites of the test group, as well as improved early wound healing indexes. When looking at the 1‐year results, the BOOST group displayed statistically significant improvements compared to the control group for all relevant clinical and radiographic parameters.

As suggested by prior literature, the site‐specific inflammatory and infectious burden should be minimized before regenerative periodontal treatment in order to decrease complications [7, 29, 30, 31]. For the present purpose, only sites with persistent deep BoP after steps I–II of therapy were selected. Two weeks after DOX application, BoP decreased to 30% in the test group and 66.7% in the control group. This reduction in interproximal tissue inflammation may account for the superior EHI results in the test group [25]. For the present investigation, a technique implying the elevation of the papilla (MIST) was chosen, with the aim to implement visibility for an adequate root debridement, which is a *conditio sine qua non* for periodontal regeneration irrespective of bone defect anatomies. However, this soft tissue management usually exposes to worse EHI compared to single flap alternatives [32, 33]. The obtained findings corroborated previous results in a smaller patient group [15] and express how reduced inflammatory local conditions and better tissue quality before surgical treatment can improve the post‐surgical healing of the interproximal tissue. This is particularly crucial for maintaining the stability of the coagulum ad preventing contamination of the site, particularly when biomaterials are used [34], and it may favor improved final results of the reconstructive therapy [35].

When analyzing the 1‐year results between groups statistically significant difference emerged for CAL gain and PPD reduction. Also, when evaluating treatment success using dichotomous definitions, a BOOST approach resulted in both significantly higher percentages of “pocket closure” [3, 27, 36] and achievement of COM [28]. These composite outcomes have become particularly relevant since they better reflect the clinical relevance of the therapy and the need for additional treatments compared to mean values [37]. Consistently, the radiographic data obtained through a standardized approach showed a statistically significant difference between the two groups for both IBD and IBA after 1 year. Overall, these findings are coherent with what is biologically and clinically plausible, which implies periodontal regeneration as an elective procedure highly sensitive to initial condition in the macro‐ and micro‐environment, as well as to intra‐operative flap management, which later reflects into a better or worse soft tissue healing [32, 33].

Among the possible site‐specific modulators of the inflammation, DOX exhibits both antimicrobial [38] and host‐modulatory properties due to its inhibition of collagenase activity through zinc ion binding [13, 39, 40]. DOX stimulates cell maturation and osteoblast differentiation, potentially enhancing the biological reservoir required for periodontal regeneration [41, 42, 43]. Furthermore, DOX is administered via a biodegradable gel, offering sustained release and effective drug concentrations at the infection site, with reduced risk of bacterial resistance [44]. Previous studies have investigated the use of DOX as an adjunct during step II therapy or for persistent/recurrent pockets during supportive periodontal care [11, 14]. Although initial clinical improvements were observed, the advantages of local DOX seemed to diminish over time, raising concerns about its value in routine clinical practice [9]. Recently, our group evaluated the short‐term benefits of DOX as a site‐specific modulator of the clinical and molecular inflammatory status prior to MIST, finding significant changes in terms of IL‐1beta, IL‐6, and MMP expression in the gingival crevicular fluid [15, 45].

Other than baseline inflammatory control, different factors may affect the results of periodontal regeneration, including systemic modifying factors, flap design, bone defect anatomy, and plaque control [4]. In order to counterbalance these variables, strict inclusion criteria were applied for patient selection. Moreover, we accounted for potential variations in flap design by only selecting cases suitable for MIST [32, 46]. Among the main limitations of this study, multiple operators performing the surgeries should be emphasized, inducing a degree of variability. Indeed, the results showed a slightly lower performance in the control group compared to some published reports where a single experienced operator performed all interventions [47, 48]. However, this variation may improve the generalizability of the results and their applicability in a broader clinical context. Moreover, the standardization of surgical technique and biomaterials (EMD + xenograft) lessen the external validity of findings, potentially diminishing the DOX impact when single‐flap procedures are utilized. A more precise selection of sites that could benefit from a BOOST approach before regenerative surgery might be achieved through a comprehensive evaluation of the molecular phenotype, which in the future could lead to a more personalized approach to treatment [41, 49]. Finally, the absence of a clinical measurement stent, the lack of evaluation of patient‐reported outcomes, and cost–benefit analyses related to the additional use of DOX represent a limit to be further addressed by future research.

## 5 Conclusion

Within the limitations of this study, it can be concluded that candidate sites for periodontal regeneration treated with a BOOST approach by local DOX showed (1) decreased clinical inflammation at 2 weeks; (2) better wound healing after surgery; and (3) significantly improved clinical and radiographic outcomes at 1 year after MIST with a combined approach involving EMD + xenograft. These results underscore once more the importance of the infective/inflammatory control prior to undergo periodontal regeneration and highlight the potential of a BOOST approach by a selective adjunctive application of an antimicrobial with anti‐collagenolytic host‐modulation properties.

## Author Contributions

M.A. contributed to conception and design, analysis, and interpretation, drafted and critically revised the manuscript. G.B. contributed to design, data acquisition and interpretation, drafted and critically revised the manuscript. V.L., N.A. and M.B. contributed to clinical procedures and drafted the manuscript. G.M.M. contributed to conception and design, and critically revised the manuscript. F.R. contributed to conception and design, data analysis and interpretation, drafted and critically revised the manuscript. All authors gave their final approval and agree to be accountable for all aspects of the work.

## Ethics Statement

The protocol was approved by the Institutional Ethical Committee of the A.O.U. Città della Salute e della Scienza of Turin (protocol number 00309/2021).

## Consent

All volunteers provided their free informed consent to participate.

## Conflicts of Interest

The authors declare no conflicts of interest.

## Data Availability

The data that support the findings of this study are listed in the main manuscript, but additional data can be obtained by the authors upon reasonable request.
